# Tilted Arch; Implementation of Additive Manufacturing and Bio-Welding of Mycelium-Based Composites

**DOI:** 10.3390/biomimetics6040068

**Published:** 2021-11-30

**Authors:** Behzad Modanloo, Ali Ghazvinian, Mohammadreza Matini, Elham Andaroodi

**Affiliations:** 1 Faculty of Architecture, University College of Fine Arts, University of Tehran, Tehran 1415564583, Iran; behzad.modanloo@ut.ac.ir (B.M.); andaroodi@ut.ac.ir (E.A.); 2 Department of Architecture, Penn State University, University Park, PA 16802, USA; 3 Faculty of Architecture and Urban Planning, University of Art, Tehran 1136813518, Iran; m.matini@art.ac.ir

**Keywords:** mycelium-based composites, additive manufacturing, bio-based materials, circular construction, digital fabrication

## Abstract

Bio-based materials have found their way to the design and fabrication in the architectural context in recent years. Fungi-based materials, especially mycelium-based composites, are a group of these materials of growing interest among scholars due to their light weight, compostable and regenerative features. However, after about a decade of introducing this material to the architectural community, the proper ways of design and fabrication with this material are still under investigation. In this paper, we tried to integrate the material properties of mycelium-based composites with computational design and digital fabrication methods to offer a promising method of construction. Regarding different characteristics of the material, we found additive manufacturing parallel to bio-welding is an appropriate fabrication method. To show the feasibility of the proposed method, we manufactured a small-scale prototype, a tilted arch, made of extruded biomass bound with bio-welding. The project is described in the paper.

## 1. Introduction

The United Nations predicts that by 2050, two-third of the world’s population will live in urban areas. Population growth in urban areas increases the demand for habitat construction. In addition, natural resources are dwindling, which has resulted in a search for sustainable and renewable alternatives for existing materials. One of the proposed solutions for these challenges is to work with bio-based materials. In addition to well-known bio-based materials such as bioplastics, materials made of bacteria, algae, and fungi have been increasingly interesting for design and fabrication. These alternative materials have led to the emergence of new design methods at the intersection of design, materials science, biology, arts, and crafts, which fundamentally changes the designer’s role from a passive receiver to an active material maker [1]. In the last decade, architects and designers have begun working with biologists and materials scientists to discover how to design and build using biomaterials [2].

In this regard, mycelium-based composites (MBCs) offer sustainable and biodegradable options for a wide range of design and fabrication processes, including architectural applications. The industrial potential of fungi in areas ranging from food production to medical biotechnology has been studied for a long time [3]. However, little research has been done in the field of architecture, engineering, and construction (AEC) [4]. Therefore, detailed research is needed to implement and build these biomaterials in the AEC industry. Furthermore, although MBCs offer many advantages for lightweight, sustainable materials [5], there are still challenges for using these materials, especially for large-scale production [6].

This paper presents the early stages of an interdisciplinary research project exploring the applications of mycelium-based composites in architecture as a sustainable, renewable, and biodegradable alternative material. This comprehensive research aims to introduce the best practices of using mycelium-based composites in architecture. In this paper, we tried to examine additive manufacturing as the forming technique for this material. In the first phase, a systematic material study was conducted to evaluate the different effects of substrate mixtures (sawdust, paper, sawdust + paper; with and without additives) on the growth and compressive strength of mycelium-based composite blocks. Following the material study, a recursive computational design and digital fabrication process have been done to construct a series of prototypes to show how the material works. The final prototype of this stage of our study, a tilted arch introduced as a proof of concept, is described in the last section.

## 2. Background

Fungi are a group of heterotrophic organisms that unlike plants, cannot produce their food through photosynthesis. Therefore, to survive, they feed on organic compounds as parasites or saprophytes. The cell walls of fungi are made of a substance called chitin. Chitin is a large and complex polysaccharide made from modified glucose chains. This substance has the primary role in the material characteristics of the fungi-based matter [7]. Mycelium is the mass fibrous and branched vegetative root of fungi, made of hyphae. Through the mycelium, a fungus absorbs nutrients from its environment. Hyphae first secrete enzymes into food sources that break down biological polymers of organic substrates into smaller units, such as monomers. These monomers are then adsorbed to the mycelium. Through this process, the mycelial branches bind the organic matter together and make a lightweight, foam-like material called mycelium-based composites [8].

Many factors affect mycelial growth, and by changing these factors, different MBC are obtained [9]. These factors include fungi used for inoculation, type of substrate, environmental conditions during growth, and formation and storage techniques [10]. The fungi and substrates used for MBC cultivation can change the characteristics of the resulting material by modifying the chemical and biological formation [7]. The environmental conditions, such as nutrients, temperature, relative humidity, pH, and aeration, can also affect the outcome of MBC cultivation [11]. The curing and post-processing of MBC can also influence the material characteristics of MBC. For example, different ways of pressing MBC via hot or cold pressing systems result in different material grades to increase material density and decrease porosity [12]. Therefore, scholars try to improve this material’s mechanical and durability behavior by tweaking each of these influencing parameters [13].

Various techniques can be used to form this material and fabricate architectural elements with MBCs [14]. One of the unique features of this material is its ability to grow in molds, allowing designers to directly grow mycelium in the final desired body shape. Processing techniques such as laser cutting and hot and cold compression can also be used to achieve the shape and structure required for grown materials [15]. In addition, the outer layer of MBC, called fungal skin [8], increases the compressive strength of the material and its water repellency. Thus, the only technique used to build large-scale architectural prototypes is to form mycelium composites into molds. However, there are also attempts on smaller scales to produce MBC elements by additive [16,17] and subtractive [6] manufacturing and fabric molds [18]. Early efforts to use MBC focused more on designing blocks and panels for walls and ceilings. As a result, a limited number of architectural projects were designed and constructed using these materials.

**Figure 1:**
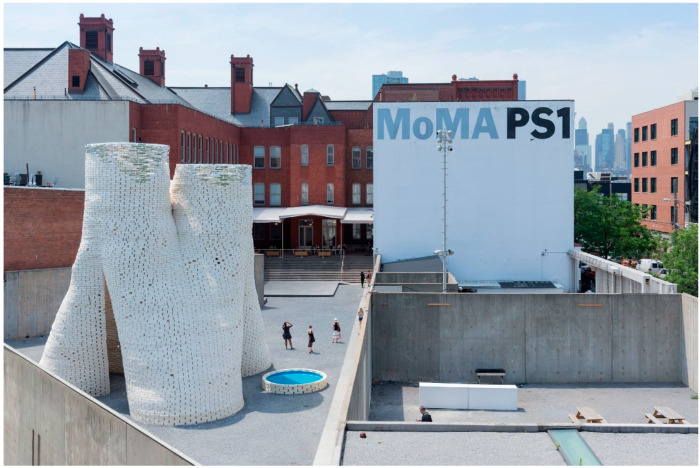
The Hy-fi Tower (Image credit: Iwan Baan).

The first architectural structure designed and built using MBC was Mycotectural Alpha fabricated by Phil Ross in 2009 as part of an art performance at the Kunsthalle Dusseldorf [19]. The Hy-Fi Tower is the largest MBC structure based on discrete elements. The tower was designed and built by David Benjamin of The Living Studios in 2014 in collaboration with Arup and Ecovative as part of the MoMA Young Architects Program. The entire 13-meter-tall structure was built using about 10,000 mycelium-based blocks (Figure 1).

**Figure 2:**
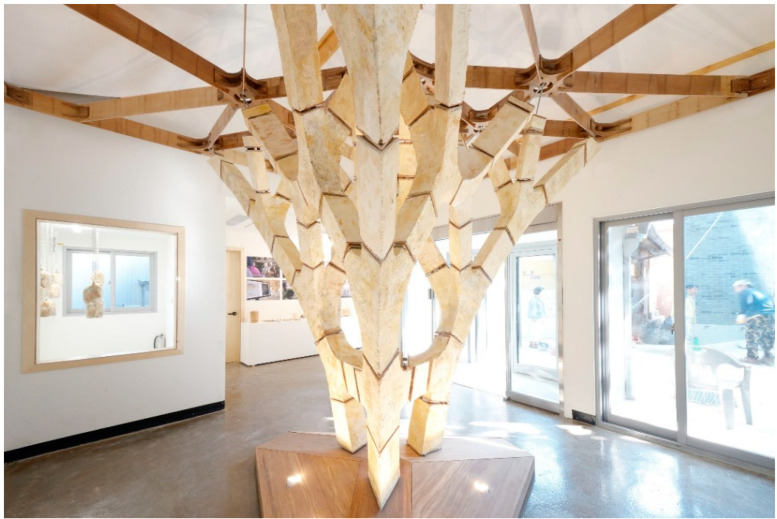
The Mycotree Pavilion (Image credit: Carlina Teteris).

BEETLES3.3 and Yassin-Areddia designed and built a temporary shell-like pavilion of wood and mycelium, called Shell Mycelium. They intended to create temporary structures without waste. Shell Mycelium was the first attempt to fabricate non-discrete structures. The triangular wooden frame of the pavilion is filled with mycelium and covered by coconut husk. As mycelium bounds the organic substrate within the growth, the result was a shell-like structure made of MBC. Mycotree is a research pavilion designed and built by the Block Research Group at ETH Zurich and the Karlsruhe Institute for Technology for the 2017 Seoul Architecture and Urban Planning Biennale (Figure 2). The structure was designed using the 3D graphic statics method [20], which allows the design of components that bear loads only under pressure.

El Monolito Micelio is the first large-scale effort of fabricating monolithic structures with MBC. For the design and fabrication of this fungi-based vault, a large formwork and a complex internal falsework have been designed, and about a ton of fresh mycelium has been cultivated [21].

Studying the outcome of these efforts and prototypes to build with MBC shows that working with this material in smaller components is more promising, as the uncertainties of coping with bio-based materials are more controllable on smaller scales. Besides, as the material’s growth depends on the presence of air, and the outer surfaces are better grown than the core of the components, working with larger pieces requires some advanced solutions and equipment to insert and distribute the air [21].

The other solution to form the MBC material in desired shapes and provide enough air to grow is the use of additive manufacturing. Compared to molding the MBC, additive manufacturing can improve fungal growth conditions, allowing faster growth and complete coverage [17]. Therefore, this method can lead to better material performance and more efficient production. In addition, it is possible to produce a complex and customized form in this process beyond what can be achieved through molding.

The first scholarly published paper about 3D printing of MBC was [22]. After the initial growth process, they opened and mixed the biomass material with additives to make it extrudable. Next, they added water and psyllium husk powder to the mixture. This powder prevents the separation of the solid phase from the liquid. In the final stage, they printed the material with a 2040 wasp printer, in the dimensions of 10 cm by 10 cm by 2 cm. Then they put the printed piece in a sterile package away from direct sunlight to grow the second stage for five days. In the final stage, the pieces were placed in a heater for four hours at 95 °C to stop the fungal growth. The results showed that, like in formworks, the fungi could not grow in all printed parts, and most of the growth took place in the exterior parts. The detailed quality differences of various material mixtures have been covered in [23]. In another research, scholars used shredded bamboo fibers and chitosan as the primary materials of MBC [24]. Bamboo fibers provide the nutrients needed for mycelial growth. The fungus used in this study is *Ganoderma lucidum*, a common fungus with fast growth and white roots. Chitosan is a biopolymer derived from chitin, the main constituent of crustaceans’ shells extracted from food waste. When chitosan is dissolved in a mildly acidic liquid medium, it forms a gel that acts as a physical stabilizer to increase the material’s efficiency and make extrusion possible.

**Figure 3:**
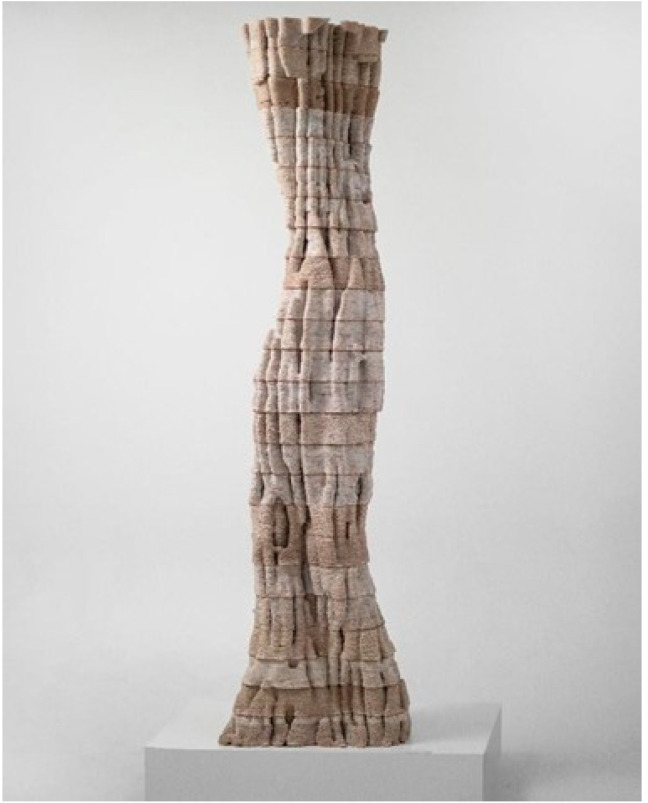
The Pulp Faction Prototype (Image credit: Ana Goidea).

One of the prototypes of 3D-printed MBC is the Pulp-Faction project [17]. This project demonstrates the challenges and potentials of additive fabrication of MBC (Figure 3). The authors used wood, pulp, and kaolin clay in combination with water to make printable pulp. Wood and paper pulp formed the bulk of the material and served to feed the fungi. The composite also contains materials that put solid and liquid components together in a cohesive manner. This study used two types of fungi, *Byssomerulius corium*, and *Gloeophyllum*, both wood decomposers. The inoculation took place in two stages, the first stage before printing and the next stage after printing. When the growth reached the final stage, the printed parts were dried to stop the decomposition process. Next, they printed the composite using a Vormvrij Lutum v4 printer. The results showed that the final volume shrinks by 30%. A set of the aluminum grid was used as a base for printing and coating to minimize the resulting distortion. The grid secured the position of the first and last layers, limiting the vertical contraction.

**Figure 4:**
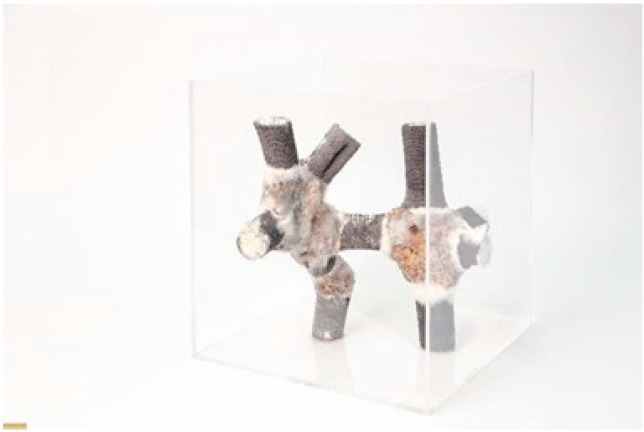
The Bar-node Structure Fabricated with Mycera (Image credit: Julian Jauk, Hana Vasatko, Lukas Gosch).

In another prototype, mixed clay and mycelium have been used to prepare a novel form of printable material called Mycera [25]. They mixed these two constituents with various proportions to enable different outcome materials. They fabricated a bar-node prototype in which the bar elements are more rigid composites, while the nodes are made of mixtures that can bind better (Figure 4).

Another line of research concentrates on using additive manufacturing of soil and fungi for mycoremediation [16]. This study tried to find the best practice of 3D printing soil-based structures and using mycelium as a remediating agent for weakened soil environments. Studying the outcome of these efforts and prototypes to print MBC shows that taking advantage of additive manufacturing for working with the MBC material enables many possibilities to enhance the forming and growth process. 3D printing is one of the best fabrication methods to increase manufacturing speed and create free forms to enable a higher strength due to geometry with a certain amount of MBC material. First, however, it is crucial to find the proper ways of printing this material due to its material characteristics.

## 3. Methodology

In order to find the best practices of using MBC for architecture, we took advantage of using Material-driven design (MDD) methodology. MDD, which aims to design by considering the limitations and affordances of the materials [1]. The MDD addresses the challenges of using this uncertain bio-based material and supports the design process when a particular material is the starting point in the design process. Relying on the concept of material experience, the MDD emphasizes that the designer’s journey of material properties and experiments leads to a broader view of material applications. The MDD method offers four main steps: (1) understanding the material, (2) creating a material experience landscape, (3) material manifestation experience patterns, and (4) product design [1].

**Figure 5:**
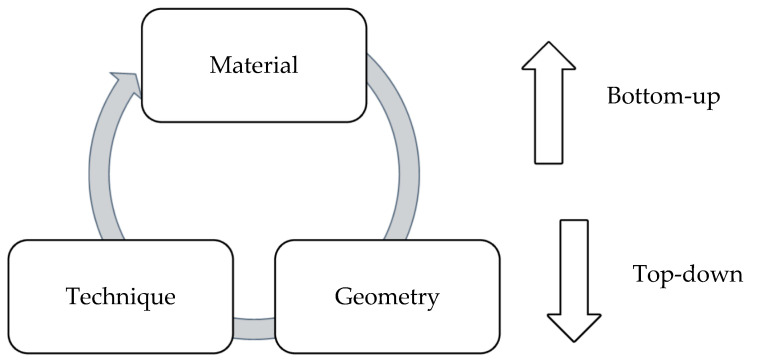
The recursive framework defined to work with novel materials/techniques (adapted from [2]).

While working with novel materials and modifying the material properties, the desired forms and geometries and the techniques to achieve these forms are also necessary [2]. These interdependent factors make us use a recursive framework with material, geometry, and technique in which each stage informs the other two stages for the subsequent iterations (Figure 5). The other important factor in this recursive study method is starting with smaller scales and scaling up the prototypes for the geometry and technique stages [26]. It means that we must consider a bottom-up part of study for enhancing our material characteristics, parallel to a top-down method for enabling our geometries and techniques with the material of choice. Therefore, we mapped the MDD on our framework, which matches the first two steps of MDD with the bottom-up part of our framework and the next two steps with the top-down stage.

### 3.1. Understanding the Material and Material Exploration

For the first two stages of MDD, we studied the literature about MBC and started an experiment with the material. The first critical decision in MBC production is the mixture of the substrate. The substrate can be composed of any organic matter if the fungus can decompose it. The substrates that are selected to produce these materials can be divided into three general categories:

- The wood waste consists of sawdust produced in carpentry—municipal waste like paper, cardboard, egg combs, and paper cups.
- Agricultural waste usually refers to the severed stems of post-harvest plants, often incinerated and producing CO_2_ in the air. Substances such as wheat straw, rice, barley, sugarcane pulp bagasse, oilseed pulp, and sesame pulp fall into this category.

Initially, to measure the growth rate of the fungus in different substrates, several organic substrates such as shredded cardboard (smaller than 5 mm), shredded paper (smaller than 5 mm), sawdust (1 to 3 mm), and chopped straw (1 to 3 mm) were selected for inoculation testing.

The other influencing parameter is the fungal species to cultivate the MBC. According to the literature, among the fungi that grow well on wood-based products, *Pleurotus* and *Ganoderma* show tremendous growth compared to other fungi [7]. Therefore, due to the abundance and fast growth of the former, we chose *Pleurotus ostreatus* (oyster mushroom), with 10% of the dry weight content, for our experiments. We bought the oyster mushroom spawns grown on wheat grains from a local provider in Tehran, Iran. As the optimized relative humidity for the cultivation of *P. ostreatus* is around 65–70%, we mixed our soaked substrates with distilled water to maintain this relative humidity.

Fungi are heterotrophic organisms that need foreign nutrients to grow. Carbon and nitrogen are the two primary nutrients needed for fungi growth. When the environment cannot provide the fungi with nutrients, additives might help [9]. We added wheat bran, with 7% of dry weight content, to help the growth process.

The MBC cultivation process requires proper sterilization to achieve good results and prevent contamination by other organisms. Sterilization is needed for both the substrate and the growth environment. For this purpose, all equipment was disinfected by soaking in 70% ethanol (Kimia Alcohol Zanjan, Zanjan, Iran) for 30 min. For the substrate sterilization, we heated the treatments in autoclave bags for 45 min at 121 °C. After inoculation, the mycelium must be maintained under controlled light, temperature, and humidity conditions to ensure sustained growth. Optimal temperature and humidity conditions vary significantly depending on the type of fungus used. However, most species grow around 25–35 °C [7].

For the initial experiments on the substrates, we cultivated the mixtures for 14 days in autoclave bags and then moved to our sample formworks for the second growth phase. Next, the samples were grown for another 14 days, then unmolded, dried for 24 h, and heated at 90 °C for 6 h.

The criterion for choosing the best growth was the visual test regarding the amount of mycelium covering the substrate on the surface of cultivated samples. After comparing the surfaces, a cross-section was cut from each sample to observe the growth of mycelium. Observations have shown that mycelium often grows in places that are in contact with oxygen. However, as predicted, shallow mycelium growth was seen inside the samples. Therefore, as the third stage of the MDD method, we decided to increase the surface contact with oxygen via 3D printing instead of molding.

In order to enable extrusion of MBC material, gelling agents are needed. These additives, when dissolved in a liquid phase as a colloidal mixture, form a homogeneous paste. Most of them are organic hydrocolloids or inorganic hydrophilic substances. Examples of these substances are tragacanth, pectin, starch, carbomer, sodium alginate, and gelatin [27]. In the literature related to the additive manufacturing of MBC, psyllium husk and chitosan have been used as gelling agents [22,23,27,28].

The gelling agents used in this study are entirely herbal and not only do not interfere with the growth of the fungus but also act as an additive to help the growth process. We experimented with several types of additives, such as Persian gum, Arabic gum, guar gum, and psyllium husk, with different proportions during the material studies. We tried to extrude straight lines of MBC that are smooth, well-grown, and with the minimum shrinkage after drying. 

**Figure 6:**
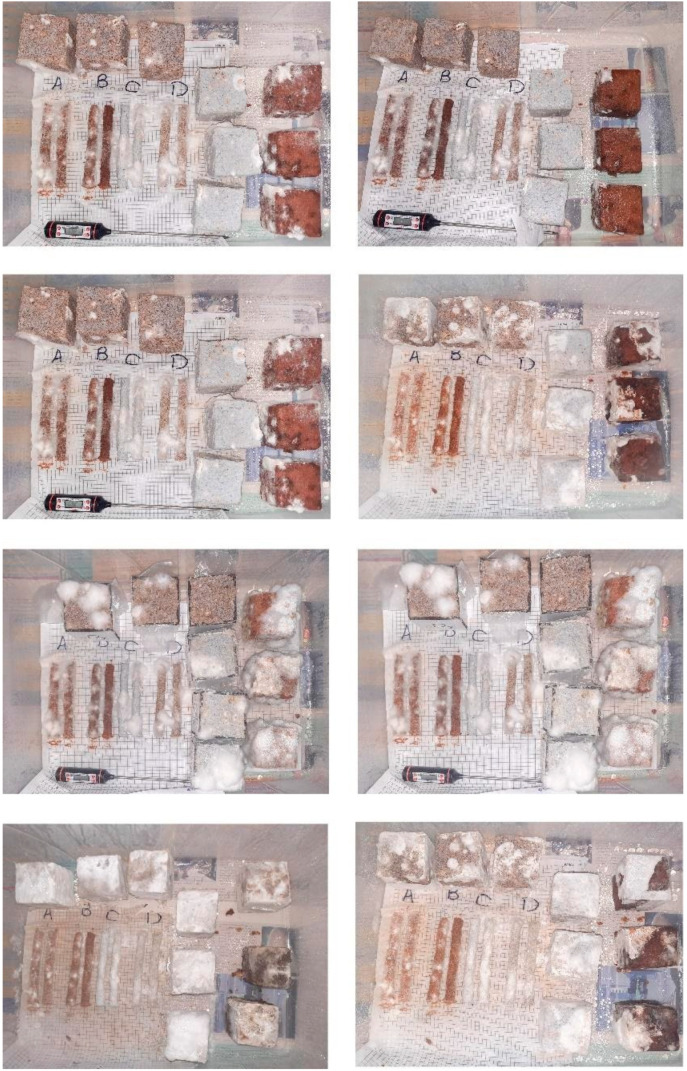
Initial material tests for examining treatments’ cultivation and gelling agents’ performance (Image credit: Authors).

Regarding the results of the cultivation and gelling agent tests, we selected two substrates and two gelling agents for printing: paper as an example of wood products and sawdust as a wood waste substrate. We chose to proceed with Arabic gum and guar gum, as they showed more consistent extruded lines and they are less expensive than other options. The most crucial characteristic of these gelling agents is that they hydrate rapidly in cold water to produce highly viscous solutions. Guar gum, exceptionally when fully hydrated, produces a homogeneous viscous colloidal mixture that is a thixotropic rheological system. Like other gums, the viscosity of guar gum depends on time, temperature, concentration, pH, and the stirring protocol [29]. Figure 6 shows parts of the initial material tests.

As the gelling agents adsorb water to make the paste homogeneous, the amount of water added to the substrate increases compared to the treatments formed with formworks. Table 1 shows the characteristics of the treatment mixtures for the second set of tests. In the second set of tests, we experimented with the mechanical behavior of different material treatments. 

**Figure 7:**
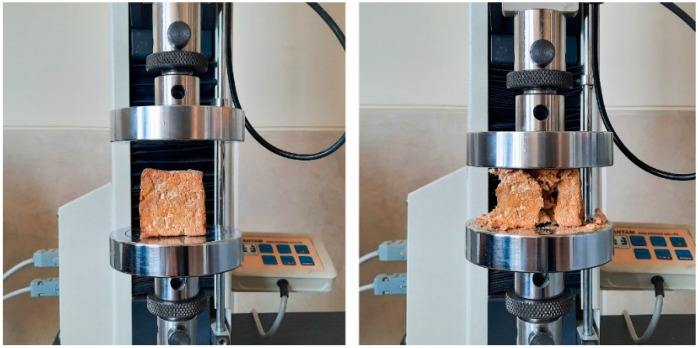
The Compressive Strength Test (Image credit: Authors).

To test the mechanical strength of these material treatments, we used a UTM machine for loading (Figure 7). First, we formed three 5 × 5 × 5 cm^3^ cubes from each treatment and loaded them at the rate of 3 mm/min. Then, examining the behavior of each sample, we drew stress-strain diagrams to obtain the yield stress and the Young modulus of each mixture.

The results of these tests showed that the paper substrate could be a suitable option for 3D printing of MBC due to its better mechanical behavior. Table 2 depicts the results of these tests. The results related to the gelling agents showed that for Arabic gum, more content is required than guar gum, while they behave the same in printability. Also, the samples cultivated with guar gum showed plastic behavior while Arabic gum showed the brittle fracture. Thus, we used paper as a substrate for the following stages of the research, with guar gum as the gelling agent. We used the same time frame for the cultivation for this stage, with 14 days in bags and 14 days in the sample formworks, followed by drying for one day and heating for 6 h at 90 °C.

### 3.2. Material Manifestation

**Figure 8:**
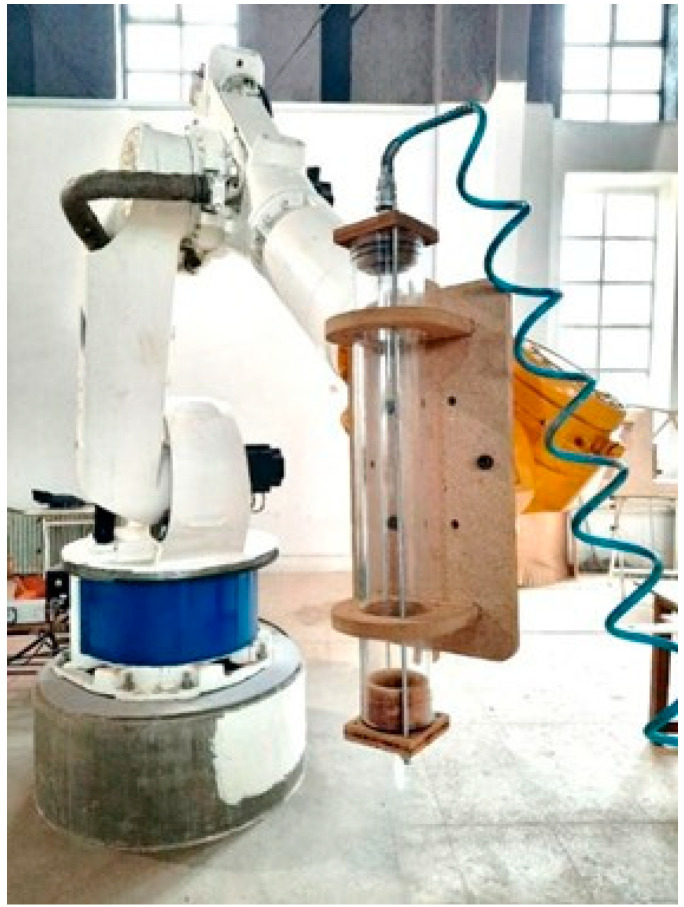
The Fabricated Extruder (Image credit: Authors).

Due to the lack of access to the market extruders, and the need for having an extruder that can be decontaminated for bio-based materials, we fabricated an extruder from scratch (Figure 8).

**Figure 9:**
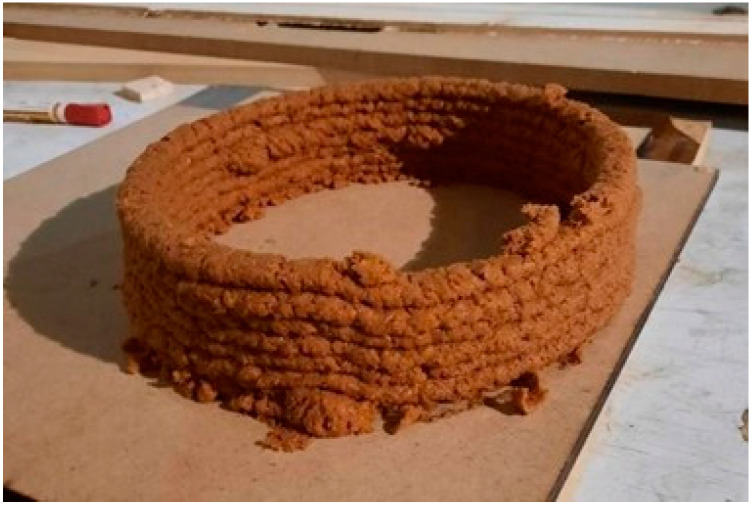
The initial extrusion experiment (Image credit: Authors).

After fabricating the extruder, we defined an experimental printing session to inquire about the best extrusion scenario. We printed several cylinders with 30 cm diameter with 5 mm to 9 mm nozzles (Table 3). We observed that the paste comes out of the smaller nozzles inconsistently and irregularly. This result showed that the low nozzle diameters do not work with the paste because oyster mushroom spawn grows on wheat and form larger particles. The best scenario happened with the 9-mm nozzle (Figure 9).

After the extrusion, the printed materials become cylindrical due to the shape of the nozzle. We tested several samples of 100 × 9 mm to inquire about the deformation after printing. When dried, the parts were measured, and no change in their dimensions was found, although they became lighter and more porous. A slight increase in cross-section was observed in layered printing due to weight accumulation in the lower layers.

### 3.3. Product Design

**Figure 10:**
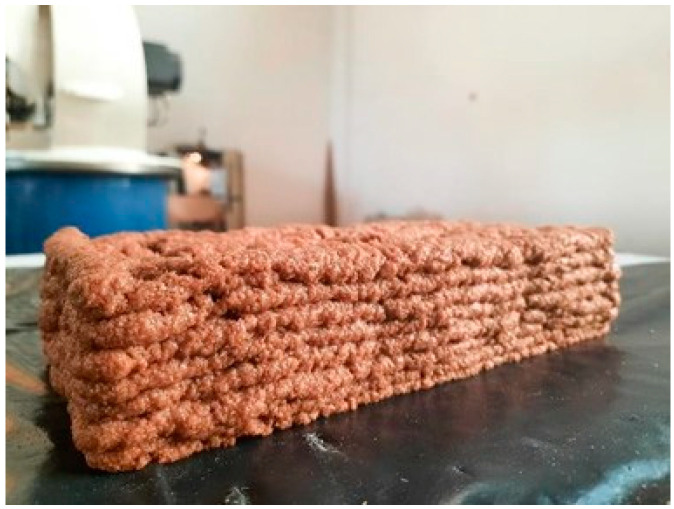
The extruded lattice brick (Image credit: Authors).

In order to take advantage of using the MBC material and additive manufacturing together, various criteria must be met. First, as the material works in compression and has little tensile strength [2,30], forms that can bear the load in a compressive manner are favorable. Second, from another perspective, additive manufacturing enables a high surface-to-volume ratio, so it is vital to design a form that can offer this feature. Finally, the limiting parameter for us, with the equipment that we fabricated, was the size of the printable object. Therefore, we started by printing diverse lattice bricks (Figure 10).

**Figure 11:**
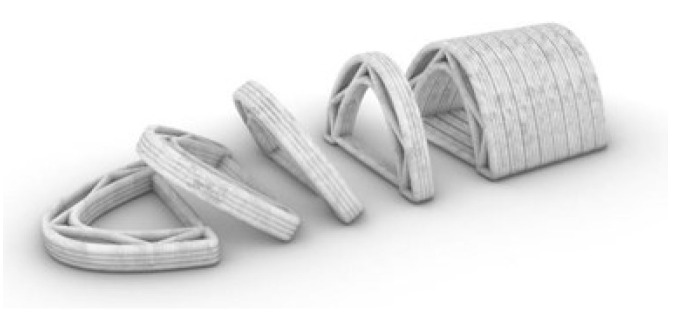
The concept design of the extruded vault (Image credit: Authors).

The results showed us a negligible difference between the mechanical strength of the lattice bricks and molded regular bricks of the same size. Thus, we decided to examine the catenary as the simplest compression-based form to better use the material and technology. We generated catenary forms within the computational environment to find the proper form to print an arch. We intended to fabricate a vault with the repetition of continuously printed arches. After extrusion and material cultivation, the arches will be tilted 90 degrees and become a vertical vault (Figure 11).

**Figure 12:**
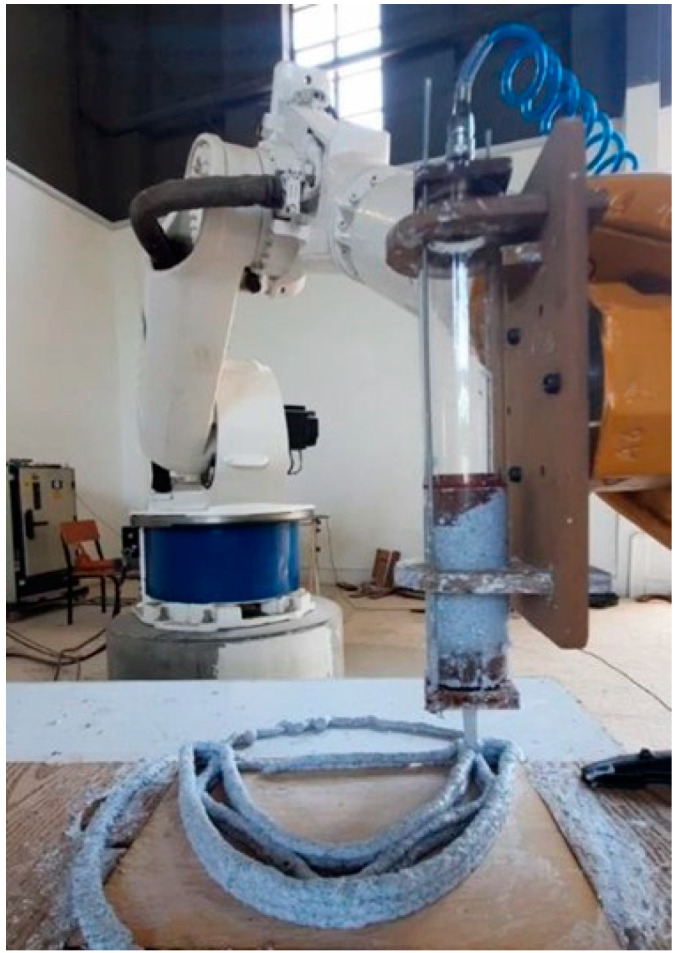
The printing session of one of the arches (Image credit: Authors).

In order to extrude the generated catenary form, it must consist of one continuous line. For this purpose, we defined a polyline that ends where it starts and then moves to the upper layer. After that, the layers are repeated as many as desired. Then, using a PRC plugin, the path is converted to a G-Code for the robotic arm connected to the extruder and printed (Figure 12).

**Figure 13:**
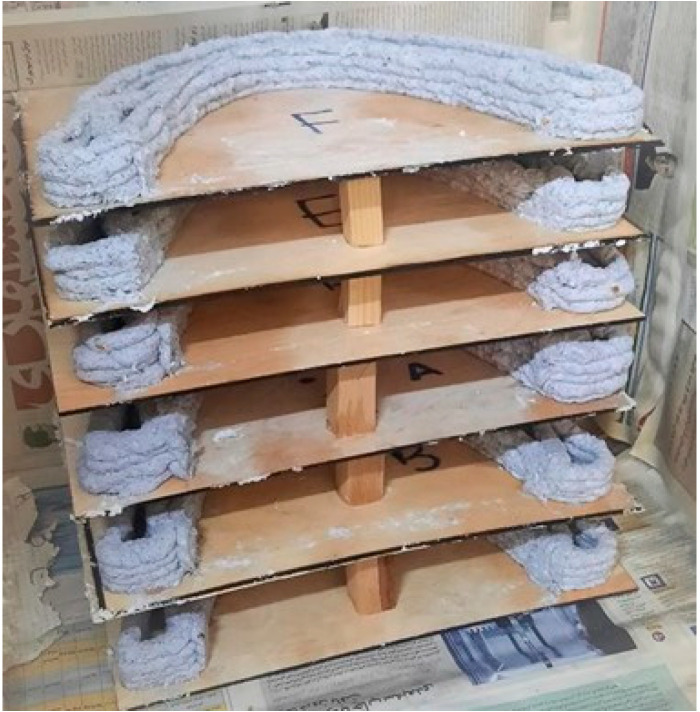
The printed arches growing in the chamber (Image credit: Authors).

We printed six similar arches with the material grown for two weeks in the bags and left them in the chamber to grow for five weeks (Figure 13).

**Figure 14:**
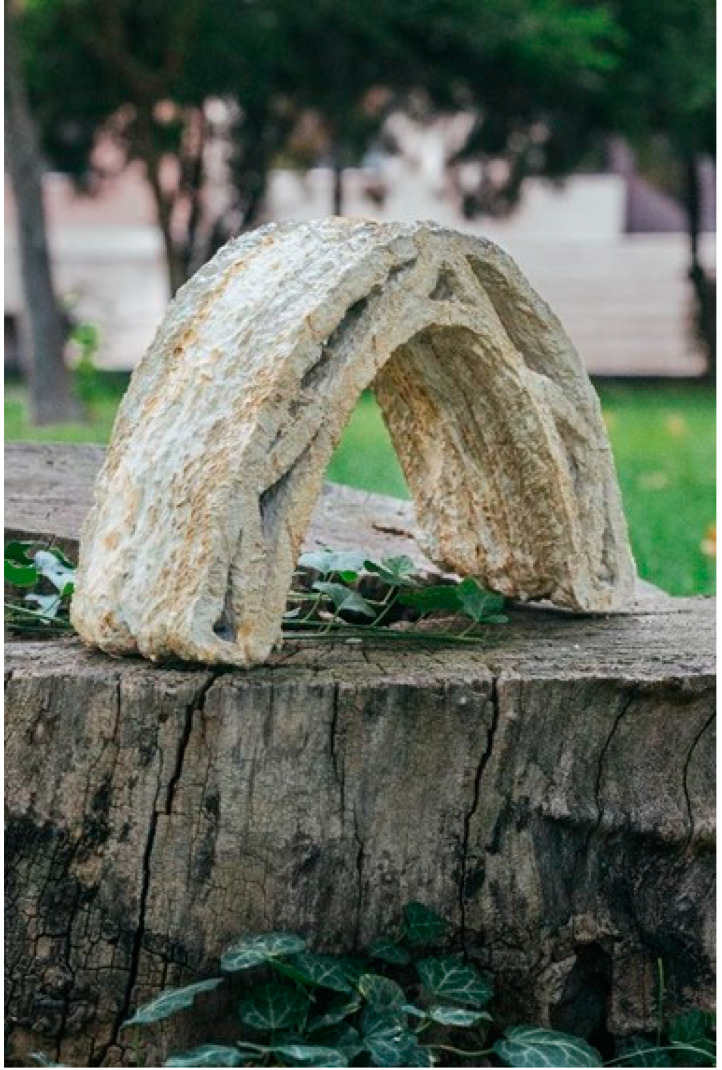
The extruded vault made of MBC material bound by bio-welding (Image credit: Authors).

After this period, regarding the ability of the living material, we put the arches on top of each other to enable the bio-welding. This feature of the material lets us bind the material without adding external binders or connections. After seven days, the arches were well-bound together and formed a vault (Figure 14).

## 4. Result and Discussion

The primary purpose of this study was to integrate the MBC material and the design computation and digital fabrication to enable the proper use of this bio-based material in the architectural context. Regarding the material characteristics of the MBC, utilizing this lightweight and quasi-weak material in a compressive manner was intended. Fabricating a catenary form that enables bearing the loads only in compression addresses this challenge. Unlike other catenary arches made with the MBC material formed with pipe-like formworks, our work is 3D printed. This fabrication method let us first fabricate with little waste due to eliminating formworks commonly made of inorganic materials such as plastics. Second, regarding the flexibility of forms that can be extruded, in comparison with molding, we could access a form with voids and divisions that make the outcome more mechanically strong, with less material and weight. Finally, as discussed in the paper, the extrusion also helped with the growth of the MBC material, as the surface-to-volume ratio is higher than the traditional molding process.

**Figure 15:**
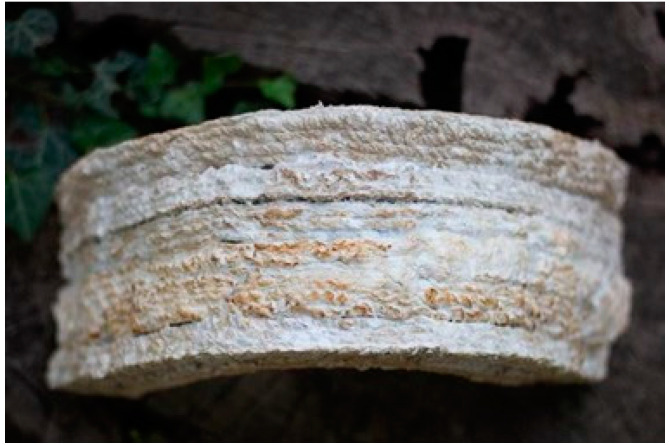
The Bio-welded Arches (Image credit: Authors).

In addition to the mentioned outcome, in this project, we could take advantage of the bio-welding of the living mycelium for the first time (Figure 15). The concept of bio-welding enables the binding of the material without using external binding agents. This feature can minimize the need for dry or wet joints and mortars in the architectural context.

## 5. Conclusions

As studied in this paper, mycelium-based composites are potential materials to substitute conventional matter in the architectural context. They offer light weight, regenerative, and compostable material features that could help address some challenges of temporary structures in the future. In this study, we aimed to inquire about the proper ways of fabrication with this material regarding its deficiencies in mechanical behavior and its compliance with additive manufacturing. We used shredded paper, oyster mushroom, wheat bran, and guar gum to prepare a treatment mixture that can be extruded and offers sufficient mechanical strength. The results showed that we could fabricate compression-only forms with this material by generating intelligent forms and geometries and using the bio-welding ability. The future challenges related to this research are scaling up the process of additive manufacturing of the MBC material and generating other innovative geometries for incremental printing of the bio-based material.

## Figures and Tables

**Table 1:** Mixture Properties for the First set of Experiments.

Treatment	Substrate	Gelling Agent	Water Content (g)
A	Sawdust (1 to 3 mm)	Guar Gum (10 g)	500
B	Sawdust (1 to 3 mm)	Arabic Gum (10 g)	500
C	Shredded Paper (>5 mm)	Guar Gum (30 g)	500
D	Shredded Paper (>5 mm)	Arabic Gum (30 g)	500

**Table 2:** Material Characteristics of Tested Mixtures.

Treatment	Yield Stress (KPa)	Young Modulus (MPa)	Behavior
A	171.44	19.1	Plastic
B	167.68	18.2	Brittle
C	524.14	41.4	Plastic
D	536.27	78.3	Brittle

**Table 3:** The Extrusion Properties of Different Nozzles.

Nozzle Size (mm)	5	6	7	8	9
Step (mm)	4	4	6	6	9
Pressure (bar)	2.2	2.1	1.8	1.7	1.7
Speed (mm/s)	100	100	100	100	100
Fresh Thickness (mm)	10	12	14	16	18
Dried Thickness (mm)	9	10	12	14	16

## Data Availability

Data is available upon request.
